# Supplementary material for: Modeling glioblastoma heterogeneity as a dynamic network of cell states
Source: Mol Syst Biol. 2021 Sep 16;17(9):e10105. doi: 10.15252/msb.202010105 (PMC8444284; doi:10.15252/msb.202010105)
Supplement: Supplementary file 5 — Source Data for Figure 3 [file MSB-17-e10105-s001.zip › Figure3A_sourcedata/GSEA_3065/nosigdb_state1.GseaPreranked.1622718409535/DOROSZKO_U3013MG_CLUSTER_2.html]

Details for gene set DOROSZKO\_U3013MG\_CLUSTER\_2[GSEA]

|  || Dataset | state1 |
| Phenotype | NoPhenotypeAvailable |
| Upregulated in class | na\_pos |
| GeneSet | DOROSZKO\_U3013MG\_CLUSTER\_2 |
| Enrichment Score (ES) | 0.5032977 |
| Normalized Enrichment Score (NES) | 1.976136 |
| Nominal p-value | 0.0 |
| FDR q-value | 2.8190605E-4 |
| FWER p-Value | 0.004 |
Table: GSEA Results Summary

  

Fig 1: Enrichment plot: DOROSZKO\_U3013MG\_CLUSTER\_2      
 Profile of the Running ES Score & Positions of GeneSet Members on the Rank Ordered List

  

| PROBE | GENE SYMBOL | GENE\_TITLE | RANK IN GENE LIST | RANK METRIC SCORE | RUNNING ES | CORE ENRICHMENT || 1 | UBE2S |  |  | 19 | 0.634 | 0.0301 | Yes |
| 2 | TUBA1C |  |  | 24 | 0.619 | 0.0610 | Yes |
| 3 | PTTG1 |  |  | 40 | 0.520 | 0.0858 | Yes |
| 4 | TUBB4B |  |  | 67 | 0.429 | 0.1048 | Yes |
| 5 | UBE2C |  |  | 125 | 0.340 | 0.1162 | Yes |
| 6 | MT2A |  |  | 161 | 0.317 | 0.1286 | Yes |
| 7 | CDKN3 |  |  | 178 | 0.307 | 0.1424 | Yes |
| 8 | CKS2 |  |  | 195 | 0.297 | 0.1558 | Yes |
| 9 | BIRC5 |  |  | 202 | 0.295 | 0.1701 | Yes |
| 10 | DTYMK |  |  | 205 | 0.292 | 0.1847 | Yes |
| 11 | POP7 |  |  | 236 | 0.282 | 0.1959 | Yes |
| 12 | JPT1 |  |  | 248 | 0.277 | 0.2088 | Yes |
| 13 | CKS1B |  |  | 346 | 0.242 | 0.2110 | Yes |
| 14 | PTGES3 |  |  | 359 | 0.237 | 0.2218 | Yes |
| 15 | YWHAH |  |  | 365 | 0.237 | 0.2332 | Yes |
| 16 | ALYREF |  |  | 371 | 0.234 | 0.2446 | Yes |
| 17 | PHF19 |  |  | 415 | 0.223 | 0.2514 | Yes |
| 18 | LSM5 |  |  | 448 | 0.216 | 0.2590 | Yes |
| 19 | CACYBP |  |  | 458 | 0.214 | 0.2689 | Yes |
| 20 | CDC20 |  |  | 469 | 0.212 | 0.2786 | Yes |
| 21 | BCL7C |  |  | 489 | 0.208 | 0.2872 | Yes |
| 22 | RHEB |  |  | 491 | 0.207 | 0.2976 | Yes |
| 23 | HDGF |  |  | 512 | 0.203 | 0.3058 | Yes |
| 24 | AURKA |  |  | 550 | 0.196 | 0.3119 | Yes |
| 25 | TPX2 |  |  | 556 | 0.194 | 0.3212 | Yes |
| 26 | CCNB1 |  |  | 583 | 0.189 | 0.3280 | Yes |
| 27 | ANP32B |  |  | 622 | 0.183 | 0.3333 | Yes |
| 28 | ILF2 |  |  | 729 | 0.167 | 0.3309 | Yes |
| 29 | CENPN |  |  | 764 | 0.163 | 0.3356 | Yes |
| 30 | KRBOX1 |  |  | 788 | 0.158 | 0.3412 | Yes |
| 31 | PGP |  |  | 852 | 0.151 | 0.3424 | Yes |
| 32 | CDCA2 |  |  | 859 | 0.150 | 0.3493 | Yes |
| 33 | HMGB3 |  |  | 907 | 0.144 | 0.3517 | Yes |
| 34 | ANLN |  |  | 941 | 0.141 | 0.3555 | Yes |
| 35 | CBR3 |  |  | 954 | 0.139 | 0.3613 | Yes |
| 36 | SAC3D1 |  |  | 976 | 0.137 | 0.3660 | Yes |
| 37 | CEP55 |  |  | 986 | 0.135 | 0.3719 | Yes |
| 38 | LMNB2 |  |  | 991 | 0.134 | 0.3783 | Yes |
| 39 | CDCA3 |  |  | 994 | 0.134 | 0.3849 | Yes |
| 40 | RPA3 |  |  | 1039 | 0.129 | 0.3869 | Yes |
| 41 | HNRNPH3 |  |  | 1054 | 0.127 | 0.3919 | Yes |
| 42 | TK1 |  |  | 1056 | 0.127 | 0.3982 | Yes |
| 43 | TROAP |  |  | 1134 | 0.119 | 0.3963 | Yes |
| 44 | NUDT1 |  |  | 1144 | 0.118 | 0.4013 | Yes |
| 45 | MAGOH |  |  | 1179 | 0.114 | 0.4036 | Yes |
| 46 | BCL2L12 |  |  | 1197 | 0.113 | 0.4076 | Yes |
| 47 | CDKN2D |  |  | 1213 | 0.111 | 0.4116 | Yes |
| 48 | CCNB2 |  |  | 1232 | 0.110 | 0.4153 | Yes |
| 49 | CENPU |  |  | 1249 | 0.108 | 0.4192 | Yes |
| 50 | DDX39A |  |  | 1255 | 0.108 | 0.4241 | Yes |
| 51 | CENPW |  |  | 1278 | 0.106 | 0.4272 | Yes |
| 52 | KIF4A |  |  | 1303 | 0.103 | 0.4299 | Yes |
| 53 | UACA |  |  | 1320 | 0.101 | 0.4334 | Yes |
| 54 | ACTL6A |  |  | 1333 | 0.100 | 0.4372 | Yes |
| 55 | CKAP2L |  |  | 1359 | 0.099 | 0.4396 | Yes |
| 56 | MAD2L1 |  |  | 1394 | 0.095 | 0.4409 | Yes |
| 57 | PLK1 |  |  | 1403 | 0.095 | 0.4449 | Yes |
| 58 | DHFR |  |  | 1413 | 0.094 | 0.4488 | Yes |
| 59 | UBE2T |  |  | 1440 | 0.093 | 0.4508 | Yes |
| 60 | ZWILCH |  |  | 1449 | 0.092 | 0.4546 | Yes |
| 61 | DEPDC1 |  |  | 1478 | 0.090 | 0.4563 | Yes |
| 62 | H2AFX |  |  | 1479 | 0.090 | 0.4609 | Yes |
| 63 | HMMR |  |  | 1480 | 0.090 | 0.4654 | Yes |
| 64 | TYMS |  |  | 1501 | 0.089 | 0.4679 | Yes |
| 65 | PPP2CA |  |  | 1535 | 0.087 | 0.4689 | Yes |
| 66 | CENPE |  |  | 1545 | 0.086 | 0.4723 | Yes |
| 67 | HMGB2 |  |  | 1570 | 0.085 | 0.4741 | Yes |
| 68 | DNAJC9 |  |  | 1594 | 0.083 | 0.4759 | Yes |
| 69 | SKA2 |  |  | 1599 | 0.082 | 0.4797 | Yes |
| 70 | KIF23 |  |  | 1638 | 0.080 | 0.4798 | Yes |
| 71 | PBK |  |  | 1651 | 0.079 | 0.4825 | Yes |
| 72 | CDC27 |  |  | 1688 | 0.076 | 0.4826 | Yes |
| 73 | ANP32E |  |  | 1706 | 0.075 | 0.4847 | Yes |
| 74 | SPDL1 |  |  | 1731 | 0.073 | 0.4859 | Yes |
| 75 | NUSAP1 |  |  | 1736 | 0.073 | 0.4892 | Yes |
| 76 | PIMREG |  |  | 1777 | 0.071 | 0.4886 | Yes |
| 77 | CENPA |  |  | 1788 | 0.070 | 0.4912 | Yes |
| 78 | PRC1 |  |  | 1794 | 0.070 | 0.4942 | Yes |
| 79 | CCNA2 |  |  | 1835 | 0.068 | 0.4935 | Yes |
| 80 | CKLF |  |  | 1837 | 0.068 | 0.4968 | Yes |
| 81 | EXOSC8 |  |  | 1851 | 0.067 | 0.4989 | Yes |
| 82 | KIF22 |  |  | 1858 | 0.067 | 0.5017 | Yes |
| 83 | USP1 |  |  | 1921 | 0.064 | 0.4985 | Yes |
| 84 | ZWINT |  |  | 1923 | 0.064 | 0.5016 | Yes |
| 85 | TACC3 |  |  | 1949 | 0.063 | 0.5022 | Yes |
| 86 | RACGAP1 |  |  | 1994 | 0.060 | 0.5007 | Yes |
| 87 | SAP30 |  |  | 2022 | 0.059 | 0.5009 | Yes |
| 88 | DLGAP5 |  |  | 2028 | 0.058 | 0.5033 | Yes |
| 89 | GMNN |  |  | 2097 | 0.055 | 0.4990 | No |
| 90 | NCAPD2 |  |  | 2121 | 0.054 | 0.4994 | No |
| 91 | ECT2 |  |  | 2150 | 0.053 | 0.4992 | No |
| 92 | CDCA8 |  |  | 2166 | 0.052 | 0.5003 | No |
| 93 | CENPH |  |  | 2215 | 0.051 | 0.4979 | No |
| 94 | KIF2C |  |  | 2224 | 0.050 | 0.4996 | No |
| 95 | SMC4 |  |  | 2308 | 0.047 | 0.4934 | No |
| 96 | TRIP13 |  |  | 2314 | 0.047 | 0.4952 | No |
| 97 | PSMD10 |  |  | 2327 | 0.046 | 0.4963 | No |
| 98 | PRR11 |  |  | 2329 | 0.046 | 0.4985 | No |
| 99 | CENPM |  |  | 2348 | 0.045 | 0.4990 | No |
| 100 | CDC25B |  |  | 2370 | 0.044 | 0.4990 | No |
| 101 | FEN1 |  |  | 2420 | 0.043 | 0.4961 | No |
| 102 | POC1A |  |  | 2424 | 0.042 | 0.4980 | No |
| 103 | GPSM2 |  |  | 2477 | 0.040 | 0.4946 | No |
| 104 | FAM83D |  |  | 2491 | 0.040 | 0.4953 | No |
| 105 | BTG3 |  |  | 2514 | 0.039 | 0.4950 | No |
| 106 | KNSTRN |  |  | 2515 | 0.039 | 0.4970 | No |
| 107 | MYBL2 |  |  | 2549 | 0.038 | 0.4955 | No |
| 108 | SPAG5 |  |  | 2566 | 0.038 | 0.4958 | No |
| 109 | GTSE1 |  |  | 2703 | 0.033 | 0.4834 | No |
| 110 | DBF4 |  |  | 2751 | 0.031 | 0.4801 | No |
| 111 | MKI67 |  |  | 2760 | 0.031 | 0.4808 | No |
| 112 | CKAP2 |  |  | 2763 | 0.031 | 0.4822 | No |
| 113 | PSMC3IP |  |  | 2766 | 0.031 | 0.4836 | No |
| 114 | ARHGAP11A |  |  | 2814 | 0.030 | 0.4802 | No |
| 115 | NEK2 |  |  | 2834 | 0.030 | 0.4798 | No |
| 116 | BUB1 |  |  | 2888 | 0.028 | 0.4757 | No |
| 117 | LSM8 |  |  | 2892 | 0.028 | 0.4768 | No |
| 118 | KIF20A |  |  | 2897 | 0.028 | 0.4778 | No |
| 119 | ASF1B |  |  | 2929 | 0.027 | 0.4759 | No |
| 120 | CIP2A |  |  | 3010 | 0.025 | 0.4689 | No |
| 121 | TMPO |  |  | 3093 | 0.023 | 0.4616 | No |
| 122 | FOXM1 |  |  | 3117 | 0.022 | 0.4603 | No |
| 123 | OIP5 |  |  | 3357 | 0.017 | 0.4364 | No |
| 124 | PARPBP |  |  | 3380 | 0.016 | 0.4350 | No |
| 125 | SKA3 |  |  | 3384 | 0.016 | 0.4355 | No |
| 126 | CDK1 |  |  | 3396 | 0.016 | 0.4352 | No |
| 127 | SPC24 |  |  | 3404 | 0.016 | 0.4353 | No |
| 128 | PCLAF |  |  | 3474 | 0.014 | 0.4289 | No |
| 129 | AC026401.3 |  |  | 3542 | 0.013 | 0.4226 | No |
| 130 | SHCBP1 |  |  | 3637 | 0.011 | 0.4134 | No |
| 131 | TTK |  |  | 3724 | 0.009 | 0.4050 | No |
| 132 | KHDRBS1 |  |  | 3753 | 0.009 | 0.4026 | No |
| 133 | SMTN |  |  | 3761 | 0.009 | 0.4023 | No |
| 134 | HJURP |  |  | 3796 | 0.008 | 0.3992 | No |
| 135 | REEP4 |  |  | 4020 | 0.004 | 0.3763 | No |
| 136 | NCAPH |  |  | 4028 | 0.004 | 0.3758 | No |
| 137 | HIRIP3 |  |  | 4163 | 0.001 | 0.3620 | No |
| 138 | SGO2 |  |  | 4184 | 0.001 | 0.3600 | No |
| 139 | CDCA5 |  |  | 4222 | 0.000 | 0.3562 | No |
| 140 | NCAPG |  |  | 4261 | -0.000 | 0.3523 | No |
| 141 | NUF2 |  |  | 4342 | -0.002 | 0.3441 | No |
| 142 | WEE1 |  |  | 4385 | -0.003 | 0.3398 | No |
| 143 | G2E3 |  |  | 4397 | -0.003 | 0.3388 | No |
| 144 | MELK |  |  | 4558 | -0.006 | 0.3226 | No |
| 145 | CENPK |  |  | 4697 | -0.007 | 0.3087 | No |
| 146 | SPC25 |  |  | 4740 | -0.008 | 0.3048 | No |
| 147 | AURKB |  |  | 4764 | -0.008 | 0.3028 | No |
| 148 | BUB3 |  |  | 4828 | -0.010 | 0.2968 | No |
| 149 | CAPS |  |  | 4880 | -0.011 | 0.2920 | No |
| 150 | RAD21 |  |  | 4916 | -0.011 | 0.2890 | No |
| 151 | MND1 |  |  | 4982 | -0.012 | 0.2829 | No |
| 152 | KIFC1 |  |  | 5017 | -0.013 | 0.2800 | No |
| 153 | TEDC1 |  |  | 5046 | -0.013 | 0.2778 | No |
| 154 | MXD3 |  |  | 5096 | -0.014 | 0.2734 | No |
| 155 | KIF20B |  |  | 5169 | -0.015 | 0.2667 | No |
| 156 | BUB1B |  |  | 5187 | -0.015 | 0.2658 | No |
| 157 | KIF14 |  |  | 5262 | -0.016 | 0.2589 | No |
| 158 | HP1BP3 |  |  | 5264 | -0.016 | 0.2597 | No |
| 159 | CCDC34 |  |  | 5298 | -0.017 | 0.2571 | No |
| 160 | KPNA2 |  |  | 5461 | -0.020 | 0.2413 | No |
| 161 | SOGA1 |  |  | 5465 | -0.020 | 0.2420 | No |
| 162 | ITGB3BP |  |  | 5717 | -0.024 | 0.2173 | No |
| 163 | CLSPN |  |  | 5848 | -0.026 | 0.2051 | No |
| 164 | RTKN2 |  |  | 5868 | -0.027 | 0.2045 | No |
| 165 | RFC3 |  |  | 6030 | -0.030 | 0.1894 | No |
| 166 | DIAPH3 |  |  | 6103 | -0.031 | 0.1835 | No |
| 167 | RRM2 |  |  | 6214 | -0.033 | 0.1738 | No |
| 168 | NDC80 |  |  | 6240 | -0.034 | 0.1729 | No |
| 169 | CENPQ |  |  | 6262 | -0.034 | 0.1725 | No |
| 170 | KIF11 |  |  | 6477 | -0.038 | 0.1523 | No |
| 171 | KNL1 |  |  | 6527 | -0.039 | 0.1492 | No |
| 172 | NCAPD3 |  |  | 6797 | -0.045 | 0.1237 | No |
| 173 | SGO1 |  |  | 6841 | -0.046 | 0.1215 | No |
| 174 | CENPF |  |  | 6902 | -0.047 | 0.1177 | No |
| 175 | MASTL |  |  | 6931 | -0.048 | 0.1173 | No |
| 176 | LMNB1 |  |  | 7166 | -0.055 | 0.0958 | No |
| 177 | EZH2 |  |  | 7260 | -0.057 | 0.0891 | No |
| 178 | CCP110 |  |  | 7307 | -0.058 | 0.0873 | No |
| 179 | CKAP5 |  |  | 7381 | -0.060 | 0.0828 | No |
| 180 | MNS1 |  |  | 7531 | -0.064 | 0.0706 | No |
| 181 | RRM1 |  |  | 7556 | -0.065 | 0.0714 | No |
| 182 | POLR2A |  |  | 7582 | -0.066 | 0.0722 | No |
| 183 | CDKN2C |  |  | 7660 | -0.068 | 0.0677 | No |
| 184 | NEURL1B |  |  | 7667 | -0.069 | 0.0705 | No |
| 185 | TOP2A |  |  | 7678 | -0.069 | 0.0730 | No |
| 186 | C21orf58 |  |  | 7804 | -0.074 | 0.0638 | No |
| 187 | ASPM |  |  | 7821 | -0.074 | 0.0659 | No |
| 188 | XPO1 |  |  | 7863 | -0.076 | 0.0655 | No |
| 189 | PHIP |  |  | 8201 | -0.089 | 0.0351 | No |
| 190 | RAD51AP1 |  |  | 8227 | -0.091 | 0.0371 | No |
| 191 | MIS18BP1 |  |  | 8274 | -0.093 | 0.0371 | No |
| 192 | NSD2 |  |  | 8351 | -0.097 | 0.0342 | No |
| 193 | FAM111A |  |  | 8359 | -0.097 | 0.0383 | No |
| 194 | CDK5RAP2 |  |  | 8496 | -0.105 | 0.0296 | No |
| 195 | KIF15 |  |  | 8542 | -0.108 | 0.0304 | No |
| 196 | SYPL1 |  |  | 8546 | -0.108 | 0.0355 | No |
| 197 | ESCO2 |  |  | 8566 | -0.109 | 0.0391 | No |
| 198 | GGH |  |  | 8643 | -0.114 | 0.0370 | No |
| 199 | SMC2 |  |  | 8721 | -0.119 | 0.0351 | No |
| 200 | CBX5 |  |  | 8780 | -0.124 | 0.0353 | No |
| 201 | HIST1H4C |  |  | 8820 | -0.126 | 0.0377 | No |
| 202 | NUCB2 |  |  | 8850 | -0.128 | 0.0412 | No |
| 203 | ORC6 |  |  | 8888 | -0.133 | 0.0441 | No |
| 204 | FANCI |  |  | 8917 | -0.136 | 0.0481 | No |
| 205 | ATAD2 |  |  | 8948 | -0.138 | 0.0520 | No |
| 206 | PSRC1 |  |  | 8973 | -0.141 | 0.0566 | No |
| 207 | ATAD5 |  |  | 9000 | -0.142 | 0.0611 | No |
| 208 | TMSB15A |  |  | 9079 | -0.150 | 0.0607 | No |
| 209 | LBR |  |  | 9264 | -0.174 | 0.0504 | No |
| 210 | BRCA1 |  |  | 9600 | -0.258 | 0.0289 | No |
Table: GSEA details [plain text format]

  

Fig 2: DOROSZKO\_U3013MG\_CLUSTER\_2: Random ES distribution      
 Gene set null distribution of ES for **DOROSZKO\_U3013MG\_CLUSTER\_2**
